# Bioactive Herbal Extracts of Traditional Chinese Medicine Applied with the Biomaterials: For the Current Applications and Advances in the Musculoskeletal System

**DOI:** 10.3389/fphar.2021.778041

**Published:** 2021-10-28

**Authors:** Haotao Li, Rongjie Wu, Haiyang Yu, Qiujian Zheng, Yuanfeng Chen

**Affiliations:** ^1^ Department of Orthopedics, Guangdong Provincial People’s Hospital, Guangdong Academy of Medical Sciences, Guangzhou, China; ^2^ Shantou University Medical College, Shantou, China; ^3^ Research Department of Medical Science, Guangdong Provincial People’s Hospital, Guangdong Academy of Medical Sciences, Guangzhou, China

**Keywords:** traditional Chinese medicine, herbal extracts, orthopedics, musculoskeletal tissue engineering, biomaterials

## Abstract

Traditional Chinese medicine (TCM) has demonstrated superior therapeutic effect for musculoskeletal diseases for thousands of years. Recently, the herbal extracts of TCM have received rapid advances in musculoskeletal tissue engineering (MTE). A literature review collecting both English and Chinese references on bioactive herbal extracts of TCM in biomaterial-based approaches was performed. This review provides an up-to-date overview of application of TCMs in the field of MTE, involving regulation of multiple signaling pathways in osteogenesis, angiogenesis, anti-inflammation, and chondrogenesis. Meanwhile, we highlight the potential advantages of TCM, opening the possibility of its extensive application in MTE. Overall, the superiority of traditional Chinese medicine turns it into an attractive candidate for coupling with advanced additive manufacturing technology.

## 1 Introduction

Musculoskeletal tissues, composed of bone, cartilage, tendon, ligament, and skeletal muscle, demonstrate poor recovery ability. In fact, the unsatisfactory regeneration of severely damaged tissue often causes pains, joint instability, and even disabilities which remains a tricky problem for surgeons. Although autologous tissue grafts, allografts, or xenografts have been broadly applied in the clinic, they have several limitations, including donor site morbidity and poor plasticity in terms of shape and structure. Furthermore, allografts and xenografts are at risk of transmitting infectious agents or even being rejected (Campana et al., 2014).

Musculoskeletal tissue engineering (MTE) emerged as a promising solution to surmount the limitations of auto- and allografts. Current strategies involve the application of tissues grafts with embedded growth factors to accelerate tissues healing. However, advanced molecules are frequently associated with high production costs or deleterious side-effects, limiting wide applicability, and therapeutic efficacy in clinical practice (Campana et al., 2014). In an effort to discover effective strategies for musculoskeletal tissues regeneration, much focus has been put on the pursuit of natural-based products owing to their availability, cost-effectiveness, and biological activity.

Traditional Chinese medicine (TCM) are becoming increasingly popular in musculoskeletal tissue regeneration. As an indispensable component of complementary and alternative medicine, TCM has evolved over thousands of years and still plays an important role in human health (Campana et al., 2014; Deng et al., 2014; Li et al., 2014). Over the last 3 decades, the application of scientific methodology has partly elucidated the underlying mechanisms of some herbal treatments. The potent bioactivity of some traditional therapies was revealed in recent years, owing to alternative therapies rigor to the study. For example, the 2015 Nobel Prize was awarded for discovering an antimalarial medicine extracted from TCM. With the improved understanding of the underlying mechanism, TCM is becoming increasingly popular in musculoskeletal tissue regeneration (Putnam et al., 2007; Liu et al., 2017; Song et al., 2018b; Zhu et al., 2021).

TCM combining conventional therapies with the MTE is a promising strategy to treat orthopedic diseases with unfavorable outcomes previously. Scientists have successfully isolated, identified, and purified various bioactive components in TCM, such as flavonoids, saponins, terpenes, alkaloids, and others (Lu and Jiang, 2012). Typically, these formulations modulate multiple signaling pathways and exert their effects on different cellular targets, enabling the treatment of orthopedic diseases with multifactorial pathogenesis. This review provides an up-to-date overview of the use of TCMs as signaling molecules and functional materials in the field of MTE, and further highlights their new and promising directions for the future.

## 2 Traditional Chinese Medicine Theory

TCM originated over 3,500 years ago, and is enjoying a resurgence in the late 20th century. The theoretical medical system of TCM was gradually developed through thousands of years of practice and refinement. It is a sophisticated set of many systematic techniques and methods including acupuncture, herbal medicine, acupressure, “qi gong”, and oriental massage.

Unlike modern medicine, TCM theory not only considers the whole person, but focuses the systematic interrelatedness of the person and nature. It is more focused on the vitalistic and synthetic aspects of humans. The major position of TCM is the balance of “yin” and “yang”. Moreover, it is through the diagnosis of “qi” disturbance that the TCM practitioner restores the “qi” balance, returning the person to a state of health.

According to TCM theories, the occurrence, development, and outcome of diseases are closely related to TCM constitutions. These constitutions form during an individual’s lifetime and are based on natural and acquired endowments (Liang et al., 2020). In addition, constitutions can not only affect disease susceptibility but also the development, outcome, and prognosis of disease. Some aspects of the orthopedic disease in modern mainstream medicine are gradually coinciding with the constitution theory of TCM. For example, the current consensus regarding the etiology and pathogenesis of osteonecrosis states that phlegm and blood-stasis constitutions are the main pathogenic factors (Yu et al., 2016). The blood-stasis constitution has a strong repair ability, but the yang-deficiency constitution has a poor repair ability and tends to collapse, requiring positive treatment. As TCM develops further, intervention measures can be established to improve TCM constitutions.

## 3 Traditional Chinese Medicine Applications in Orthopedics

Recently, the application of TCM in MTE is booming and show superior therapeutic potential. Some herbs have been proven to be beneficial to bone health, such as Eucommiae Cortex (Du-Zhong) (Qi et al., 2019), Achuranthes (Huai-niu-xi) (Zhang et al., 2018; Song et al., 2018a), Dipsaci Radix (Xu-Duan) (Ke et al., 2016), Testudinis Plastrum (Gui-ban) (Liang et al., 2016; Ren et al., 2017; Shen et al., 2018), Drynariae Rhizoma (Gu sui-bu) (Qiu et al., 2016; Song et al., 2017b), Du Huo Ji Sheng Tang (Wang et al., 2017b), and zuo Gui Wan (Lai et al., 2015). Among these TCMs, the epimedii herb and its multicomponent formulation ‘Xian Ling Gu Bao’ have icariin as their main ingredient and have been used to cure bone diseases such as osteoporosis and bone fracture (Zhang et al., 2014). In parallel, lamiophlomis rotate (Du Yi Wei) pill was used to treatment of pain in clinic, such as bone and muscle pain, joint pain, and dysmenorrhea. It has the effect of promoting blood circulation, relieving pain, stopping bleeding, and removing silt form the perspective of TCM (Li et al., 2010). Moreover, another TCM regent named salvia miltiorrhiza injection, has therapeutic effects in cardiovascular disease with the antioxidant, antibacterial, and anticoagulant properties (Wang et al., 2017b). These herbal medications were approved officially by the Chinese Food and Drug Administration and have been widely used in clinic nowadays. Various studies discussed in the following paragraphs would provide further evidence of the realistic possibility of TCM being used as a therapeutic alternative in the foreseeable future.

## 4 Multifunctional Traditional Chinese Medicine

### 4.1 Icariin

**FIGURE 1:**
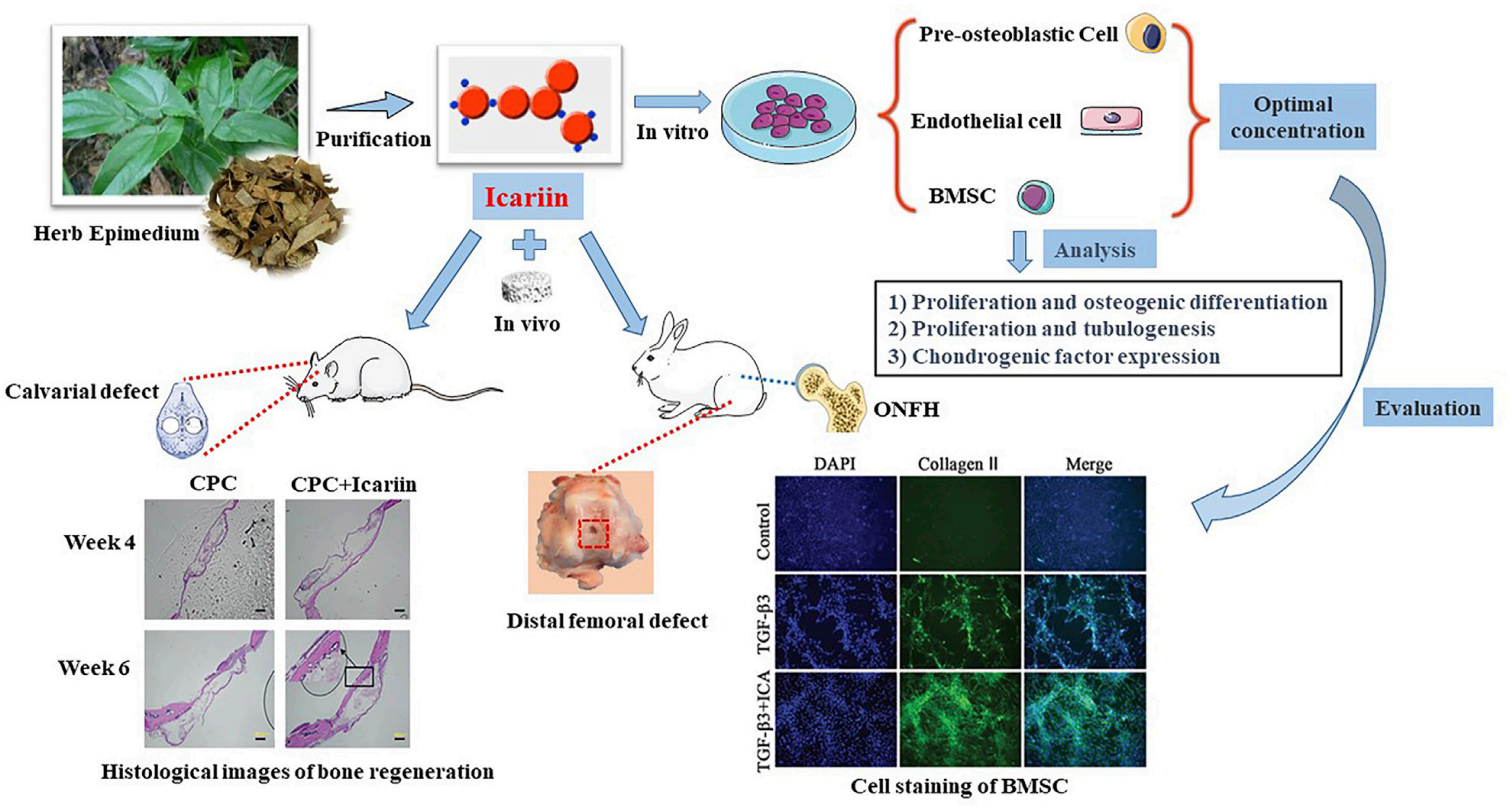
Illustration of icariin: the origin, molecular structure, and biomedical researches. Icariin is obtained from the leaves of Epimedium herb. As a widely used TCM, icariin demonstrates extensive therapeutic capacities such as osteogenic differentiation, tubulogenesis, and chondrogenic factor expression. Calvarial defect, distal femoral defect and ONFH mice/rabbits are commonly used animal models.

Icariin (Figure 1)is a prenyl flavonoid glycoside isolated from Epimedium herb, demonstrating extensive therapeutic capacities such as osteoprotective effect, neuroprotective effect, cardiovascular protective effect, anti-cancer effect, anti-inflammation effect, immunoprotective effect, and reproductive function (Li et al., 2015).

#### 4.1.1 Osteogenesis

Currently, the significant osteogenic effect of icariin made it a promising drug candidate in bone tissue engineering. As early as 2008, the osteoinductive potential of icariin on pre-osteoblastic cells was elucidated (Zhao et al., 2008). The extremely low cost of icariin and its high abundance makes it appealing for bone regeneration (Fan et al., 2011). Importantly, icariin can be steadily and locally released by using biomaterials, making it an attractive osteoinductive candidate for bone tissue engineering (Zhao et al., 2010; Fan et al., 2012; Zhang et al., 2013).

Previous studies have shown that icariin could induce osteogenic differentiation of preosteoblastic cells. For example, Zhao et al. confirmed the anabolic effect of icariin *in vivo* employing a mouse calvarial defect model. Calcium phosphate cement loaded with icariin filled in the mouse calvarial bone defect induced significant new bone formation and increased bone thickness. The study utilizing senescence-accelerated mouse models further demonstrated that icariin significantly enhanced bone formation *in vivo* (Zhao et al., 2010). Moreover, Wu et al. constructed the calcium phosphate cement scaffolds, which loaded with icariin and then implanted into the calvarial defect of the ovariectomized rats. The results demonstrated that icariin could up-regulate the expression of osteogenic and angiogenic genes in bone marrow stem cells (BMSCs) (Wu et al., 2017).

In parallel, icariin could also be loaded into porous β-tricalcium phosphate (β-PTCP) ceramic disks to enhance the ability of calcium phosphate-based biomaterials for bone defect repair. β-TCP has been employed extensively as a substitution material for bone defect repair (Park et al., 2014; Sándor et al., 2014). It was showed that loading icariin in β-TCP (Ica/β-TCP) disks had no effect on the attachment and morphology of Ros17/28 cells. However, the Ica/β-TCP disks expressed support for the proliferation and differentiation of Ros17/28 cells better compared with the β-PTCP disks. After back intramuscular implantation of rats for 3 months, no obvious osteogenic evidence was detected in β-PTCP disks, but new bone formation was observed in Ica/β-TCP disks (Zhang et al., 2011).

In addition to synthetic scaffolds, icariin could be loaded onto the natural scaffold or small intestine submucosa (ICA-SIS) to improve their osteoinductivity (Li et al., 2017). *In vitro* experiments revealed that expression of osteogenic differentiation markers (Alp, Bsp, and Ocn) was increased after treatment of ICA-SIS scaffold while no significant cytotoxicity was indicated. In an *in vivo* mouse calvarial defect model, bone regeneration was enhanced by SIS implantation at 8 weeks, compared to the control defect. These results suggest that icariin had the potential to be used for bone defect repair.

#### 4.1.2 Angiogenesis

Vascularization is considered to be a crucial step in bone formation (Wernike et al., 2010). It is reported that icariin stimulated *in vitro* endothelial cell proliferation, migration, and tubulogenesis, as well as increasing *in vivo* angiogenesis (Chung et al., 2008). In a recent study, three-dimensional (3D) printing β-TCP scaffold loaded with icariin was implanted into the steroid-induced osteonecrosis of the femoral head rabbit models. Immunohistochemical staining revealed that a higher positive rate of vascular endothelial growth factor in the composite scaffold group. This result suggested that icariin could not only protect the injured vascular endothelial cells, but also stimulate angiogenesis directly (Ji et al., 2005; Wang and Huang, 2005; Xu and Huang, 2007), and it might be the potential drugs in angiogenic therapy.

#### 4.1.3 Chondrogenesis

Hyaline articular cartilage lacks blood vessels, lymphatics, and nerves and exhibits limited self-repair ability after injury. Traditional techniques of articular cartilage repair and regeneration all have certain limitations. The development of tissue engineering technology has brought hope to the regeneration of articular cartilage. Icariin at a low dose of 0.94 G/kg was identified to have significantly promoted the proliferation of chondrocytes and enhance the secretion of glycosaminoglycan (Zhang et al., 2019). Femoral condyle from rabbits treated with icariin conditioned serum and hyaluronic acid was observed to regenerate more native cartilage and subchondral bone regeneration. *In vitro* study, ICA significantly upregulated the mRNA expression levels and protein synthesis of collagen II, aggrecan, and Sox9, which were chondrogenic differentiation markers (Wang et al., 2014).

In a recent study, BMSCs were cultivated in a self-assembling peptide nanofiber hydrogel scaffold in a chondrogenic medium for 3 weeks (Wang et al., 2018). Icariin was added to the medium throughout the culture period. The results demonstrated that icariin significantly enhanced cartilage extracellular matrix synthesis and gene expression levels of collagen II and Sox9, and additionally promoted more chondrocyte-like rounded morphology in BMSCs. In another study, icariin was added into cell-hydrogel constructs derived from neonatal rabbit chondrocytes and collagen type I (Li et al., 2012). The results showed that icariin-added cell-hydrogel constructs obviously up-regulated the expressions included aggrecan, Sox9, and collagen type II of seeded chondrocytes from 99.7 to 248%. Moreover, the icariin-loaded hydrogel improved the restoration efficiency of supercritical-sized osteochondral defects in adult rabbit model, and enhanced the integration of new-formed cartilage with subchondral bone. These results demonstrated the potential of icariin to promote a reparative response in cartilage defects and the possible application in bioactive material-based cartilage regeneration therapies.

### 4.2 Ginseng

**FIGURE 2:**
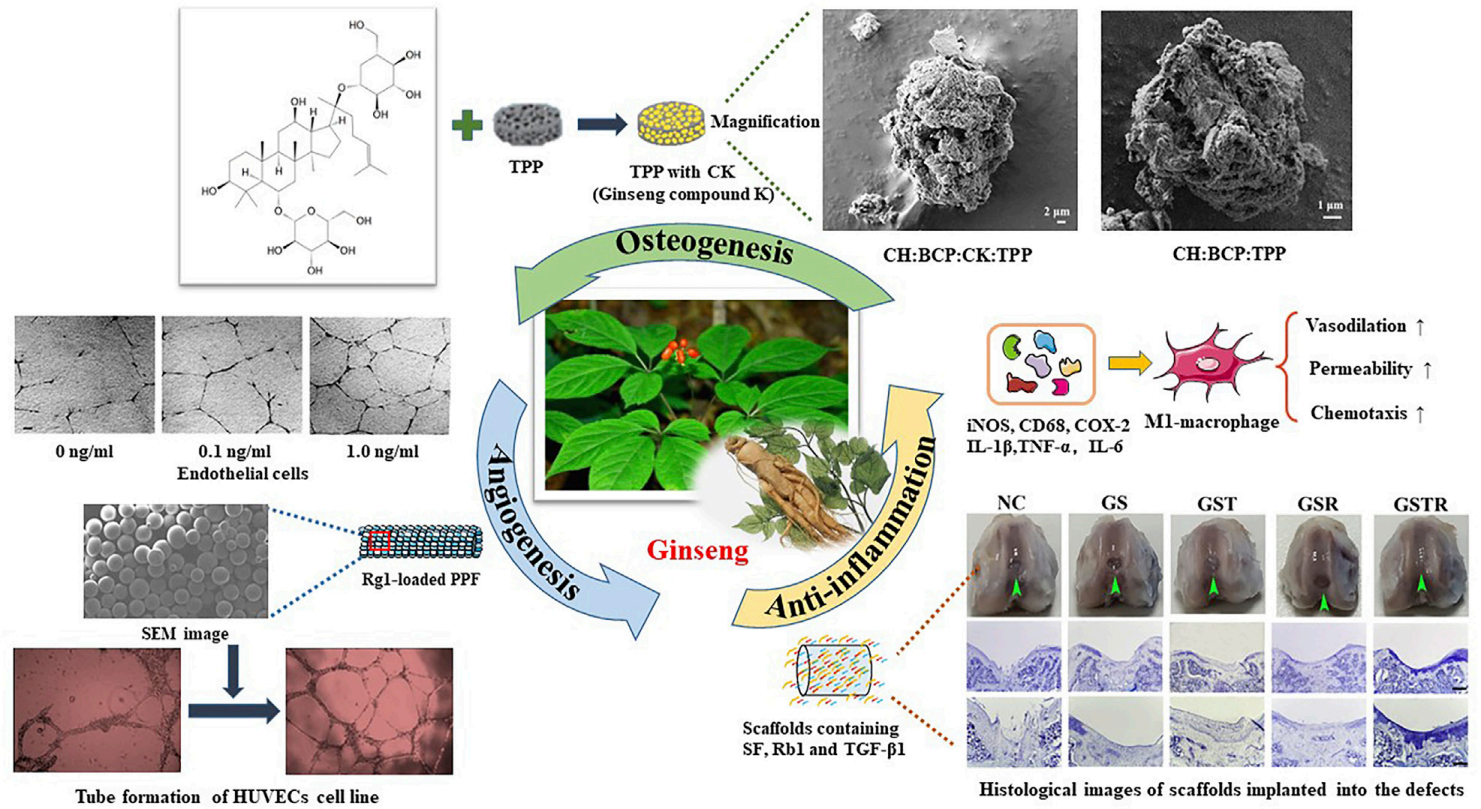
Illustration of ginseng: the origin, molecular structure, and biomedical researches. Ginsenoside is the major active ingredient in ginseng, including Rb1, Rb2, Rc, Rd, Re, Rg1, and so on. Thus far, ginseng has been frequently studied form the aspects of cells, biomaterials, and animals. It shows potent angiogenesis, osteogenesis, and anti-inflammation activity by inducing osteogenic cell proliferation, tube formation, and macrophage chemotaxis.

Ginseng (Figure 2), regards as the king of all herbs, has been used as a traditional medicine for the treatment of diseases for thousands of years in East Asian countries. The principal bioactive components of ginseng are ginsenosides such as Rb1, Rb2, Rc, Rd, Re, and Rg1, showing various anti-inflammatory, antioxidant, antibacterial, antiviral, and antifungal functions. Based on the advantages above, ginseng may provide the basis for the development of novel therapeutic agents.

#### 4.2.1 Osteogenesis

Previous studies have demonstrated that Rg1 could improve the anti-aging ability of BMSCs, which was attributed to the anti-oxidant and anti-inflammatory capacities (Hu et al., 2015; Zeng et al., 2018). Moreover, differentiation culture analysis showed that Rg1 could guide human bone marrow-derived mesenchymal stem cells (hBM-MSCs) towards osteogenic lineage while suppressing adipogenic differentiation (Wang et al., 2020c). Additionally, Hong et al. observed that Rg3 increased proliferation and suppressed senescence of hBM-MSCs (Hong et al., 2020). Suitable scaffold materials were prepared using fish scale collagen, hydroxyapatite, chitosan, and beta-tricalcium phosphate. Ginseng compound K was incorporated into the composite scaffold. *In vitro* analysis showed that the prepared scaffold was biocompatible and supported the growth of MG-63 cells (human osteosarcoma cells), and therefore has potential as an alternative approach for bone regeneration (Muthukumar et al., 2016).

#### 4.2.2 Angiogenesis

On the other hand, Rg1 has been proven to have estrogen-like activity (Chan et al., 2002). It is well known that estrogen could modulate angiogenesis *via* effects on endothelial cells (Morales et al., 1995). Therefore, it suggests that Rg1 may be used to stimulate angiogenesis.

Based on the above potential, Rg1 was encapsulated into biodegradable poly propylene fumarate (PPF) microspheres to facilitate osteogenesis (Salarian et al., 2016). In the presence of Rg1 within the dose range of 1–32 μg/ml, elongated and robust capillary-like networks were formed *in vitro* attributed to a greater number of cells compared with the control, indicating the angiogenic activity of Rg1. The previous results suggested that Rg1 could be a novel group of angiogenic agents with superior stability and may be used for the MTE (Liang et al., 2005).

#### 4.2.3 Anti-Inflammation

Inflammatory cytokines induced by traumatic lesions in cartilage and osteoarthritis (OA) are involved in lubricin catabolism and cartilage degeneration, further disrupting the normal homeostasis in articular cartilage to the breakdown of cartilage in the pre-OA conditions (Billinghurst et al., 1997; Elsaid et al., 2007; Goldring and Otero, 2011; Wojdasiewicz et al., 2014). Thus, it is vital to maintain a proper microenvironment for the chondrogenic differentiation of endogenous stem cells at the defected area. Ginseng has been demonstrated to have therapeutic potential in anti-inflammatory, anti-apoptosis, and neuroprotective responses (Radad et al., 2004; Hashimoto et al., 2012).

Porous, stable and biodegradable bone microsphere scaffold loading with ginseng compound was studied, and *in vitro* results indicated that the composite microspheres expressed higher osteogenic markers in rat bone marrow stem cells seeded (Thangavelu et al., 2020). In another study**,** Wu et al. designed a novel Rb1/TGF-β1 loaded silk fibroin-gelatin porous scaffold with the advantages of inflammation attenuation and chondrogenesis promotion (Wu et al., 2020). As the results showed, the scaffold promoted the chondrogenic differentiation of BMSCs and suppressed inflammation gene expression. Moreover, it effectively promoted the regeneration of hyaline cartilage of the osteochondral defects in rats. These results prove Rb1 has a great potential to maintain an anti-inflammation microenvironment for cartilage repair.

### 4.3 Naringin

**FIGURE 3:**
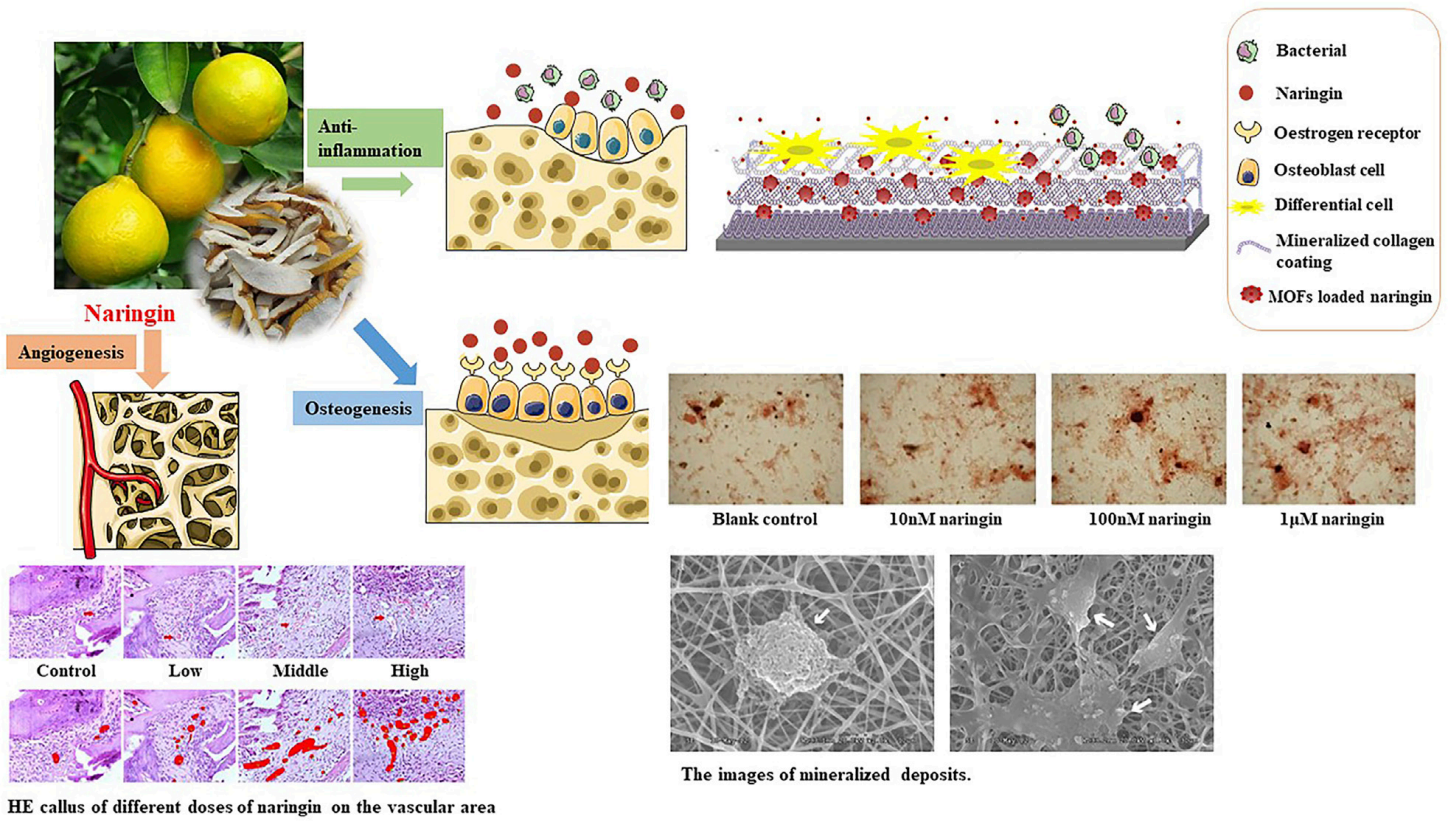
Illustration of naringin: the origin and biomedical researches. Naringin is a natural flavonoid present in several fruits of the Citrus genus. As a flavonoid with multiple therapeutic targets in orthopedic tissues, naringin exhibits osteogenic, angiogenic, and anti-inflammation effects in preclinical studies.

Gu sui-bu is a commonly used Chinese medical herb for therapeutic treatment of bone-related diseases, and naringin (Figure 3) is the main active component in Gu sui-bu. Recent research has focused on the potential applications as a bone therapeutic or as a mediator of MSC osteogenic lineage differentiation (Chen et al., 2016).

#### 4.3.1 Osteogenesis

Pang et al. confirmed naringin significantly prompted osteogenic in osteoblast-like cells via estrogen receptor-dependent pathways (Pang et al., 2010). Importantly, this study demonstrated that naringin exerts tissue-selective estrogenic effects on bone and possibly in adipose tissue, suggesting the potential antiresorptive capacity of naringin. The low bioavailability and extensive metabolism of naringin motivated researchers to explore MTE for immobilizing or protecting it from degradation and for achieving a sustained spatiotemporally controlled release to improve its therapeutic effect.

Initially, human periodontal dental ligament stem cells were seeded in a nanohydroxyapatite scaffold and cultured in a naringin-containing medium for 1 week, following by implanting into healthy mice. The transplant was harvested 8 weeks later and the naringin-treated group exhibited improved trabecular bone maturity surrounding the scaffold (Yin et al., 2015). In a recent study, naringin was incorporated in the electrospun nanoscaffold containing poly (ɛ-caprolactone) (PCL) and poly (ethylene glycol)-block-poly (ɛ-caprolactone) (PEG-b-PCL) (Ji et al., 2014). Osteoblast-nanoscaffold interactions were studied and osteoclast-nanoscaffolds response was evaluated in a mouse calvarial defect organ culture model. The results demonstrated that controlled-release naringin nanoscaffolds supported osteoblast adhesion, proliferation, differentiation, and mineralization more effectively while suppressing osteoclast formation. Alternatively, Chen et al. developed a porous biodegradable composite comprising genipin-crosslinked gelatin and β-Ca_3_(PO_4_)_2_ ceramic microparticles (GGT) mixed with naringin (10 mg/ml) (Chen et al., 2013). The potential of the composites in repairing bone defects was evaluated and compared *in vivo* by using the biological response of rabbit calvarial bone to these composites. After 8 weeks of implantation, naringin-loaded GGT composites promoted a significant deposition of new bone formation when compared with GGT controls.

#### 4.3.2 Anti-Inflammation

Moreover, previous studies have further demonstrated the antibacterial function of naringin (Tsui et al., 2008). To better exert inherent antimicrobial and pro-osteogenic effects of naringin, Yu et al. designed a multifunctional mineralized collagen coating on titanium with the aid of metal-organic framework nanocrystals to control the release of naringin (Yu et al., 2017). The attachment, proliferation, osteogenic differentiation, and mineralization of mesenchymal stem cells on the coating were significantly enhanced. Meanwhile, antibacterial abilities against *Staphylococcus aureus* were also promoted.

#### 4.3.3 Angiogenesis

In addition to the properties of osteogenesis and anti-inflammation, naringin could also regulate the function of endothelial cells to promote angiogenesis in bone (Shangguan et al., 2017). Meanwhile, oral administration of naringin could improve the expression of vascular endothelial factor, further augmenting the vascularization of the callus in osteoporotic fractures in ovariectomized rats (Song et al., 2017a). Although these studies have illustrated the angiogenic activity of naringin, the application of naringin to promote vessel ingrowth is still scarce. Given the superior osteogenic, anti-inflammation, and angiogenic properties, naringin is regarded as an excellent candidate for MTE and regenerative medicine application.

## 5 Monofunctional Traditional Chinese Medicine

### 5.1 Psoralen

Psoralen is an active component in TCM Buguzhi which means ‘‘material for bone strengthening,” which has been reported to have antibacterial, anti-tumor, coronary artery broadening, and estrogen-like activity (Guo et al., 2003). It has also been reported that the extract of psoralen could promote osteoblastic differentiation as evidence of increased Alp (Xiong et al., 2003).

In order to determine whether psoralen could also increase the amount of new bone formation locally, Wong and Rabie measured the amount of new bone produced by psoralen with collagen matrix carrier grafted into bone defects in rabbits (Wong and Rabie, 2011). As the histological assessment showed, a total of 454% more new bone was present in defects grafted with psoralen in collagen matrix than those grafted with collagen matrix. There was also more amount of bone forming osteoblasts in the psoralen group than the negative control–collagen group. This comparison showed that psoralen was osteogenic when used with the collagen matrix.

### 5.2 Kaempferol

Kaempferol (Kaem) is a widespread naturally occurring flavonoid in plants and herbs. It is known for its activity in anti-inflammatory, anti-oxidant, anti-cancer, and anti-ulcer properties (Chen and Chen, 2013). In orthopedic aspects, Kaem has been reported to reduce glucocorticoid-induced bone loss and promote osteoblast differentiation (Prouillet et al., 2004; Adhikary et al., 2018). Moreover, Kaem exerts profound anti-osteoclastogenic effects by specifically antagonizing tumor necrosis factor receptor family action on bone cells, by disrupting production of osteoclastogenic cytokines from osteoblasts and attenuating osteoclast precursor cell differentiation (Pang et al., 2006).

A recent study investigating the bioactive glass scaffold loaded with Kaem showed that Kaem could support bioactivity and cell attachment (Ranjbar et al., 2021). In another study, TiO_2_ implant-immobilized Kaem could be an effective tool for bone regeneration in rats (Tsuchiya et al., 2018). The results showed that BMSCs cultured on alkali-treated TiO_2_ samples containing Kaem promoted alkaline phosphatase activity, calcium deposition, and osteogenic differentiation. The *in vivo* histological analysis revealed that Kaem stimulated new bone formation around implants.

### 5.3 Ursolic Acid

Ursolic acid (UA) is one of many ubiquitous triterpenoids in medicinal herbs. It is found throughout the plant kingdom and constitutes an integral part of the human diet (Xia et al., 2011). Pharmacological effects of UA include anti-cancer (Hsu et al., 1997), pro-differentiation (Lee et al., 1994), anti-viral (Quéré et al., 1996), and anti-invasion activities (Cha et al., 1998).

Interestingly, Lee et al. reported that UA promotes bone formation and induces bone forming activity *in vivo* (Lee et al., 2008). Furthermore, they showed that the expression of osteoblast-specific genes was enhanced after UA treatment, and that UA could induce osteoblastogenesis and mineralization of osteoblasts *in vitro*, which was associated with the activation of mitogen-activated protein kinases (MAPKs), activator protein-1 (AP-1), and nuclear factor-kappaB (NF-kB). Studies have also demonstrated that UA activates chondrocytes through the NF-kB/NLRP3 inflammasome pathway, thus preventing cartilage degeneration in osteoarthritis (Wang et al., 2020a). In consideration of the various beneficial effects of UA, it can be considered an effective treatment strategy for OA.

Mesoporous bioglass/chitosan porous scaffolds loaded with UA have been demonstrated to enhance bone regeneration (Ge et al., 2019). The as-released UA drugs from the scaffolds increased the alkaline phosphatase activity, osteogenic differentiation-related gene type I collagen, runt-related transcription factor 2 expression, and osteoblast-associated protein expression remarkably.

### 5.4 Curcumin

Curcumin, the active constituent for turmeric, is known for its antioxidant, anti-inflammatory, anticancer, and osteogenic activities. Numerous studies published in diverse *in vivo* models, such as lung inflammation, asthma, sepsis, intestinal inflammation, osteoarthritis, and psoriasis (Daily et al., 2016; Burge et al., 2019; Karimi et al., 2019; Panahi et al., 2019; Shahid et al., 2019; Zahedipour et al., 2020), documenting the anti-inflammatory properties of curcumin. However, its poor bioavailability, rapid metabolism, and rapid systemic elimination led to limiting oral efficacy in various preclinical and clinical studies. To enhance its bioavailability and to provide higher release, several scaffolds loaded with curcumin have been designed.

Cur-loaded microspheres were incorporated into a fish collagen nano-hydroxyapatite scaffold to promote bone repair under diabetic conditions (Li and Zhang, 2018). Curcumin released from the composite scaffolds lasted up to 30 days and remarkably alleviated the negative effects of diabetic serum on the proliferation, migration, and osteogenic differentiation of mesenchymal stem cells. Furthermore, tissue scaffolds containing a low concentration of curcumin could increase gene and protein expression related to osteogenesis (Jain et al., 2016). Interestingly, in another study, liposomal curcumin released from the three-dimensional printed scaffold showed significant cytotoxicity toward *in vitro* osteosarcoma cells, whereas it promoted osteoblast cell viability, and proliferation (Sarkar and Bose, 2019). Moreover, the composite hydrogel loading with Mg^2+^ and curcumin could simultaneously exert anti-inflammatory and pro-differentiation effects to accelerate rotator cuff healing (Chen et al., 2021).

Calcium silicate cements have excellent bioactivity and can induce the bone-like apatite formation (Chen et al., 2015; Huang et al., 2015). However, they have degradability and the dissolved calcium silicate can cause the inflammatory response at the early post-implantation stage. Based on these, a study designed the curcumin-loaded mesoporous calcium silicate cement to reduce the inflammatory reaction after implantation (Chen et al., 2017). As the results showed, it could inhibit the expression of TNF-α and IL-1 after inflammatory reaction induced by lipopolysaccharides and had good anti-inflammatory ability. It can provide an excellent strategy to inhibit the inflammatory response for MTE and bone regenerative medicine.

### 5.5 Thymol

Thymol is a natural product obtained from oregano leaves and is used for various purposes, such as for their antimicrobial, antioxidant, and anti-inflammatory activities (Burt et al., 2005; Liang et al., 2014). It has been shown to reduce the key mediators of inflammatory cytokines (Fachini-Queiroz et al., 2012; Amirghofran et al., 2016). The bioactive effects of thymol combined with MTE is still understudied. A study investigated the effect of thymol on osteogenesis, specifically with osteoblast, and osteoclast cells, from surface-modified Ti6Al4V with plasma sprayed hydroxyapatite coatings (Vu and Bose, 2020). Thymol shows bacterial inhibition of *Staphylococcus* epidermidis and no cytotoxic effects on osteoblast proliferation *in vitro*. Despite the scarcity of research, the potential application of thymol in combination with orthopedic biomaterials has been shown.

### 5.6 Danshen

In order to achieve better bone formation performance, it is necessary to develop some alternative agents with both osteogenesis and angiogenesis, especially for the patients with osteonecrosis of the femoral head. Danshen, or Salvia miltiorrhiza Bunge is a TCM widely used for the treatment of cardiovascular diseases by improving blood circulation and inhibiting inflammatory responses (Zhou et al., 2005). Animal studies have supported the bone protective effect of danshen, and investigation on individual active components of danshen showed a similar effect (Chae et al., 2004; Cui et al., 2011). Salvianolic acid B (SB) is the most abundant molecule isolated from the aqueous fraction of danshen (Yuan et al., 2005), and it has been proven to have the bioactivities of both angiogenesis (Lay et al., 2003; Liu et al., 2007; Tang et al., 2014; Xu et al., 2014; Guo et al., 2014a).

A recent study constructed a SB-loaded chitosan/hydroxyapatite scaffold and loaded it onto the rabbit radius defect model to evaluate the bone repair effect. The angiogenic bioactivities of the SB-loaded chitosan/hydroxyapatite scaffold were proved to be effective by *in vivo* and *in vitro* tests (Ji et al., 2019). In another study, SB was bound to the graphene oxide (GO) (Wang et al., 2020b),and silk fibroin (SF) substrates were combined with functionalized GO through the freeze-drying method. After the SF/GO/SB scaffolds were implanted in a rat cranial defect model, the defect area showed more new bone and angiogenesis than that following SF and SF/GO scaffold implantation. Lin et al. developed a bioactive composite scaffold incorporated with SB and evaluated the effects on spinal fusion models (Lin et al., 2019). Results revealed the effect of SB on new bone formation, mineral apposition rate, and vessel density within the scaffold. In summary, these studies suggested that SB could enhance bony fusion through the promotion of angiogenesis.

## 6 Others

In fact, in addition to the therapeutic potential of TCMs mentioned above, there are other interesting targets. For example, Asperosaponin VI, a natural compound isolated from the well-known traditional Chinese herb Radix Dipsaci, promotes osteoblast formation. The pharmacological study has demonstrated that Asperosaponin VI promoted MC3T3-E1 and primary rat osteoblasts proliferation, and enhanced the formation of bone nodules in osteoblast cells (Niu et al., 2011). In addition, it can promote osteogenic differentiation of adipose-derived stem cells by inducing the expression of bone-related proteins (Ocn and RUNX2, and Smad2/3 phosphorylation) (Ding et al., 2019). Another TCM called cinnamaldehyde, a bioactive cinnamon essential oil from Cinnamomum cassia, has been reported to have multipharmacological activities including anti-inflammation. Recently, researchers have found it can suppress proinflammatory cytokines secretion in rheumatology arthritis synoviocyte cells by Janus kinase/signal transducer and activator of transcription pathway (Luo et al., 2020). As the *in vivo* results showed, cinnamaldehyde ameliorated collagen-induced arthritis in rats. These findings indicate that cinnamaldehyde is a potential traditional Chinese medicine-derived, disease-modifying, antirheumatic herbal drug.

There have been many *in vitro* and animal studies provided multidimensional evidence of the efficacy and mechanisms of icariin in treating OA models. Interestingly, a recent study suggests an endocannabinoid-related pathways associated with OA pain and a hypothalamic-related mechanism involving icariin effects (Li et al., 2021). The quantitative proteomics and bioinformatics analyses confirmed that relieving OA pain might be an important mechanism involved in the effects of icariin. These findings contribute to considering icariin as a novel therapy for OA.

MSCs are multipotent stem cells and have the potential to differentiate into several cell linages, including osteoblasts, chondrocytes, adipocytes, cardiomyocytes, and endothelial cells (Ghasroldasht et al., 2014; Oryan et al., 2017). Thus, recruiting MSCs from surrounding tissues or circulation to the fracture callus is very important for bone repair and regeneration (Herrmann et al., 2015; Zhou et al., 2017). In a recent study, Zhu et al. investigated the effect of cyasterone (exact from Radix cyathulae) on MSCs migration and osteogenic differentiation *in vitro*, as well as its role in the healing of rat fractures (Zhu et al., 2021). As the results showed, cyasterone could promote the migration and osteogenesis capacities of MSCs. The fractured femurs healed faster with the treatment of cyasterone. Meanwhile, cyasterone could significantly increase the level of stromal-derived factor-1α (SDF-1α), which is one of the main chemokines of stem cells, in rats with femur fracture. Even though these findings were not combing with MTE, they enriched the function of TCM as a regenerative medicine and shed new light for the treatment of bone defects in clinical research.

## 7 Forecast

Musculoskeletal tissues damage, which can be caused by trauma, cancer, and bone disorders, poses formidable public health burdens. Although the strong evidence of the effect of herbal extracts in orthopedics and its potential when combined with biomaterials, there are still some obstacles on the application of herbal extracts in effective clinical use.

### 7.1 Current Obstacles in TCM Clinical Translation

Despite its promising therapeutic action in several pathologies, particularly in improving degenerative diseases and treating bone defect, most TCM is yet to be widely used in modern medicine. This fact is mainly related to the extensive metabolism of TCM *in vivo*, a crucial factor that limits their therapeutic efficacy. Nowadays the effects of TCM have been mainly explored *via* oral absorption; due to the poor solubility and high intestinal liver metabolism of some herbs, it showed limited oral efficacy in various preclinical and clinical studies. Additionally, the intestinal microbiota plays a crucial part in defining the bioavailability of TCM such as naringin (Cassidy and Minihane, 2017). In fact, this microbiome is characterized by substantial heterogeneity between individuals, and defines the clinical efficacy of dietary TCM ultimately.

Adding to this, another problem of studying TCM is to quantify Chinese herbs. Current Chinese herb studies focus on the identification of active components (Guo et al., 2011; Feng et al., 2014; Guo et al., 2014b), which greatly differ from those used in the clinical setting. TCM stresses compatibility when uses medications, which means that active ingredients are combined to produce a therapeutic effect, and the single active ingredient of a single drug cannot fully prove the mechanism of action. Concerning the compatibility of TCM, relevant literature remains scarce and there is a great untapped potential to be exploited for this part. Future exploration should concentrate on enhancing the bioavailability and providing higher release of TCMs.

### 7.2 Herbal Extracts and Biomaterials

On account of these limitations, there have been many *in vitro* attempts for improving bioavailability and absorption, by combining biomaterials with herbal extracts, including encapsulation in nanoparticles or microparticles. As recognized from previously highlighted studies with the natural compounds (e.g., quercetin), controlled delivery via nanocarriers can significantly improve their *in vivo* therapeutic effect (Ahmad et al., 2016). These nanocarriers can provide a sustained release profile for locally improving accumulation at the desired locations, ultimately improving the bioavailability of herbal extracts (Muhamad et al., 2018). Moreover, 3D bio-printing has enabled the fabrication of well-designed 3D constructs for use as transplants with specific shapes and features using various biomaterials and cells (Murphy and Atala, 2014). A new sustained release hydrogel scaffolds composited of mesoporous bioactive glass, sodium alginate, and gelatin were fabricated by the 3D printing technique (Wu et al., 2019). Naringin was used to prepare drug-loaded scaffolds by direct printing or surface absorption. The results showed that MG-63 cells cultured with the extract containing released drugs displayed promoted cell proliferation and the expression of osteogenesis-related genes more effectively compared with the drug-free extract.

In turn, biomaterials used in MTE should have homologous mechanical properties to maintain the morphology and function of tissues. Some herbal extracts can add specific properties to biomaterials, and therefore improves efficacy of the biomaterial. The incorporation of ginseng extract improved the physical characteristics (i.e., hydrophilicity) of PCL nanofibers, as well as the mechanical properties (Pajoumshariati et al., 2016). Although ginseng extract increased the degradation rate of pure PCL nanofibers, the porous structure and morphology of fibers did not change significantly after 42 days. Additionally, a recent study fabricated TCMs incorporated fish collagen film, which has good biocompatibility in mammalian cell growth. In this study, three types of TCMs including genistein, icariin, and naringin were used for film fabrication (Wang et al., 2021). Mechanical properties of collagen films were improved by the addition of TCMs, especially in collagen-naringin films. Furthermore, the solubility and *in vitro* biodegradation of collagen films were enhanced by the hydrophobicity and chemical interaction of TCMs with collagen. Considering the mutual effects between herb extracts and biomaterials, it can be used to investigate the effect of *in vitro* stem cell culture.

### 7.3 Conclusion

**FIGURE 4:**
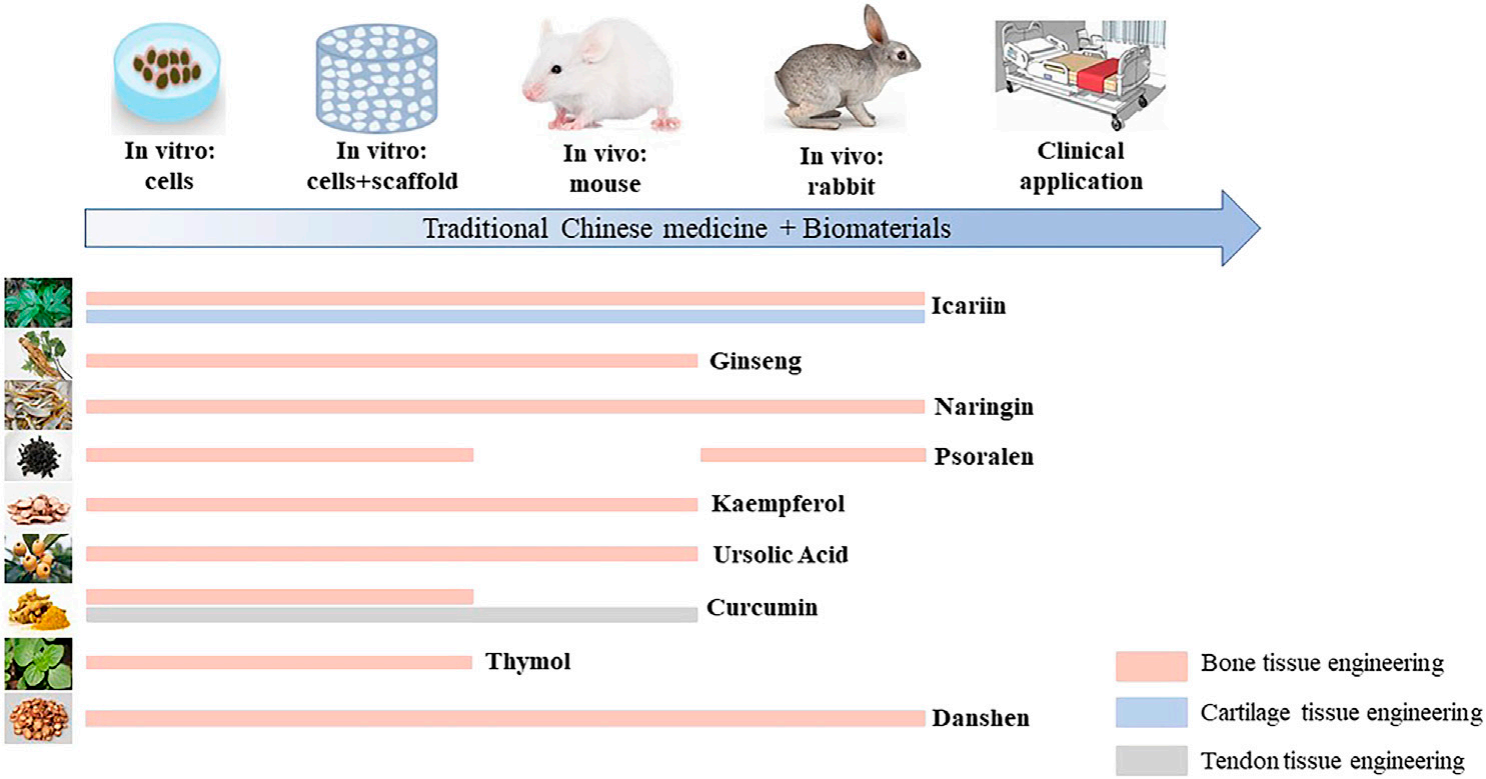
Schematic representation of the musculoskeletal application of traditional Chinese medicines.

In conclusion, TCM have triggered the enthusiasm of many researchers due to their excellent and diverse therapeutic properties. In the past few decades, the demand for safer and more suitable agents has guided researchers to explore the potential of TCM for treating chronic bone diseases. These results reveal the novel approaches toward the fabrication of MTE, which couples the advanced additive manufacturing technology with the wisdom of alternative medicine; the reported potential of TCM makes it a very attractive candidate for coupling with the advanced additive manufacturing technology (Figure 4). Most of these attempts have been effective but remain in a preclinical stage, and more efforts should be paid on *in vivo* studies. Besides, studies about the regeneration of skeletal muscle tissue are obviously less than those for hard tissues. Thus, further research and development would be necessary to ensure their safe and effective clinical use.

## Abbreviations

Alp, alkaline phosphatase; AP-1, activator protein-1; BMSCs, bone marrow stem cells; Bsp, bone sialoprotein; β-TCP, β-tricalcium phosphate; Cur, curcumin; 3D, three-dimensional; GO, graphene oxide; hBM-MSCs, human bone marrow-derived mesenchymal stem cells; ICA, icariin; Kaem, kaempferol; MAPKs, mitogen-activated protein kinases; MTE, musculoskeletal tissue engineering; NF-kB, nuclear factor-kappaB; OA, osteoarthritis; Ocn, osteocalcin; ONFH, osteonecrosis of the femoral head; PEG-b-PCL, poly (ethylene glycol)-block-poly (ɛ-caprolactone); PPF, poly propylene fumarate; SB, salvianolic acid B; SDF-1α, stromal-derived factor-1α; SF, silk fibroin; SIS, small intestine submucosa; TCM, traditional Chinese medicine; UA, ursolic acid.
